# First-trimester HbA_1c_ in relation to plasma glucose concentrations in an oral glucose tolerance test at 12 to 16 weeks’ gestation—a population-based study

**DOI:** 10.1186/s13098-024-01290-3

**Published:** 2024-02-27

**Authors:** Mervi Jokelainen, Hannu Kautiainen, Arja Nenonen, Beata Stach-Lempinen, Miira M. Klemetti

**Affiliations:** 1 Obstetrics and Gynecology, South Karelia Central Hospital, Valto Käkelän katu 1, Lappeenranta, 53130 Finland; 2 Obstetrics and Gynecology, University of Helsinki and Helsinki University Hospital, Haartmaninkatu 2, Helsinki, 00029 HUS Finland; 3 Folkhälsan Research Centre, Haartmaninkatu 8, Helsinki, 000290 Finland; 4 Primary Health Care Unit, Kuopio University Hospital, P.O. Box 100, Kuopio, FI 70029 KYS Finland; 5 Laboratory Center, South Karelia Central Hospital, Valto Käkelän katu 1, Lappeenranta, 53130 Finland

**Keywords:** Early pregnancy, Gestational diabetes, Glycated hemoglobin, Oral glucose tolerance test

## Abstract

**Background:**

Early-onset GDM often requires pharmacological treatment and is associated with adverse perinatal outcomes, but data is insufficient regarding the best methods to identify high-risk women requiring early GDM screening. The aim of this study was to analyze the diagnostic accuracy of HbA_1c_ in the prediction of (1) plasma glucose concentrations > 90th percentile in an oral glucose tolerance test (OGTT) at 12–16 weeks’ gestation; and (2) pharmacologically treated early- or late-onset GDM.

**Methods:**

HbA_1c_ was measured at 8—14 weeks’ gestation in a population-based cohort of 1394 Finnish women recruited for the Early Diagnosis of Diabetes in Pregnancy (EDDIE) study between 3/2013 and 12/2016. Information on maternal risk factors were collected at recruitment. Subsequently, a 2-hour 75 g OGTT was performed at 12—16 weeks’ gestation (OGTT1), and if normal, repeated at 24–28 weeks’ gestation (OGTT2). Early- and late-onset GDM were diagnosed using the same nationally endorsed cut-offs for fasting, 1 h- and 2 h-plasma glucose: ≥5.3, ≥ 10.0mmol/l, and/or ≥ 8.6mmol/l, respectively. In total, 52/1394 (3.7%) women required metformin or insulin treatment for GDM, including 39 women with early-onset GDM diagnosed at OGTT1 and 13 women with late-onset GDM diagnosed at OGTT2.

**Results:**

Maternal early-pregnancy HbA_1c_ ≥ 35mmol/mol (≥ 5.4%) was the best cut-off to predict fasting or post-load plasma glucose > 90th percentile in OGTT1, but its diagnostic accuracy was low [AUC (95% CI) 0.65 (0.62 to 0.69), sensitivity 0.55 (0.49 to 0.60) and specificity 0.67 (0.64 to 0.70)] both alone and in combination with other maternal risk factors. However, HbA_1c_ ≥ 35mmol/mol correlated positively with plasma glucose concentrations at all time points of OGTT1 and predicted pharmacologically treated GDM diagnosed at OGTT1 or OGTT2; AUC (95% CI) 0.75 (0.68 to 0.81), sensitivity 0.75 (0.61 to 0.86), specificity 0.64 (0.61 to 0.66).

**Conclusions:**

In our population-based cohort, early-pregnancy HbA_1c_ ≥ 35mmol/mol was positively associated with fasting and post-load plasma glucose concentrations in an OGTT at 12—16 weeks’ gestation and predicted pharmacologically-treated early- and late-onset GDM, suggesting potential utility in first-trimester identification of women at high risk of severe GDM subtypes.

**Supplementary Information:**

The online version contains supplementary material available at 10.1186/s13098-024-01290-3.

## Background

Gestational diabetes (GDM) affects up to ∼15–30% of pregnancies and is associated with short- and long-term adverse health outcomes in both the mother and child [1–3]. Obstetric and perinatal complications are particularly common in GDM subtypes with early onset and need for pharmacological treatment [4, 5]. Currently, GDM screening is usually performed between 24 and 28 weeks’ gestation, using an oral glucose tolerance test (OGTT). However, increasing evidence points towards the importance of early pregnancy metabolic milieu as a determinant of fetoplacental development and pregnancy outcomes [6–12]. A recent randomized controlled trial showed that immediate treatment of women with early-onset GDM diagnosed < 20 weeks’ gestation reduces neonatal complications [13]. Hence, practical clinical tools for the identification of women at the highest risk of developing early hyperglycemia or severe GDM requiring pharmacological treatment are needed.

Since OGTTs are laborious to perform and require maternal fasting, alternative methods, such as glycated hemoglobin (HbA_1c_) assessment, have been actively investigated for the screening of maternal hyperglycemia. Disappointingly, many studies have suggested that early-pregnancy HbA_1c_ performs poorly in the prediction of late-onset GDM diagnosed after 20 weeks’ gestation [14, 15]. In contrast, evidence regarding the utility of first-trimester HbA_1c_ assessment in the prediction of early-pregnancy maternal hyperglycemia < 20 weeks’ gestation is less abundant. In theory, HbA_1c_ – which reflects glycemic control during the preceding ∼3-month period – could be a better screening tool for the identification of women with early-pregnancy dysglycemia and severe GDM subtypes requiring pharmacological treatment, since these women are more likely to be characterized by periconceptional metabolic derangements. However, previous studies in unselected [16, 17] and high-risk populations [18–20] have yielded inconsistent results.

To our knowledge, no previous large population-based studies are available on the relationship between maternal HbA_1c_ and plasma glucose concentrations in an early-pregnancy OGTT. Hypothesizing that women with early-onset or severe GDM subtypes are most likely to have chronic disturbances of glucose metabolism leading to elevated early-pregnancy HbA_1c_ levels, we aimed to analyze 1) relationships between HbA_1c_ measured at 8 to 14 weeks’ gestation (i.e., before conventional OGTT screening) and maternal plasma glucose concentrations during a 2 h 75 g OGTT at 12 to 16 weeks’ gestation; and 2) whether HbA_1c_, alone or in combination with other maternal risk factors of GDM, predicts plasma glucose concentrations > 90th percentile in an OGTT at 12–16 week’ gestation, 3) whether early-pregnancy HbA_1c_ predicts pharmacologically treated early- or late-onset GDM in a population-based cohort of pregnant women.

## Methods

The Early Diagnosis of Diabetes in Pregnancy (EDDIE) study was implemented at South Karelia Central Hospital (SKCH), a secondary-level referral hospital in Lappeenranta, southeastern Finland, with a catchment population of ~ 133 000. SKCH is the only center providing specialist antenatal, obstetric, and neonatal care in the region, and all deliveries (~ 1000/year) in the area are managed at SKCH.

The formation of the population-based EDDIE cohort, sample collection, and clinical follow-up have been described in detail [21]. A flow chart depicting the formation of the study population for the present study is presented in Supplementary Fig. 1.

Briefly, from March 2013 to December 2016, 2305 women who booked for the first-trimester screening ultrasound examination were assessed and recruited by a trained nurse. The first-trimester screening ultrasound is offered to all pregnant women living in the South Karelia are and performed either at SKCH in Lappeenranta or at Honkaharju Hospital in Imatra. Women with pre-existing diabetes, multiple gestations, difficulties in understanding the consent forms, or oral corticosteroid medications were excluded from the recruitment. Of the invited women, 527 (22.9%) refused to participate.

At the recruitment visit, at 8–14 weeks’ gestation, data on maternal anamnestic risk factors of GDM were collected using a structured questionnaire, and a blood sample was drawn for HbA_1c_ assessment. In addition, maternal height, weight, and waist circumference (WC) were measured and recorded. WC was measured midway between the lowest ribs and the iliac crest. Waist-to-height ratio (WHtR) was calculated by dividing WC by height. Pre-gestational weight was self-reported, and it was compared by a clinician to the first weight measured in early pregnancy at the first antenatal care visit. If the difference was implausible, the pre-pregnancy weight was deleted.

1401 of the recruited women completed a 2-hour 75 g OGTT at 11.6–16.4 weeks’ gestation (OGTT1). Women with missing, incomplete, or wrongly timed OGTT1 were excluded from the final study population. Of the 1401 women with complete OGTT1 results available, 1394 women who had their HbA_1c_ measured at the recruitment visit at 8–14 weeks’ gestation composed the final study population for the present study (Supplementary Fig. 1).

GDM was diagnosed using the OGTT criteria recommended by the Finnish Current Care Guidelines (FCCG): i.e., if one or more plasma glucose concentrations (0 h, 1 h and/or 2 h) during OGTT1 exceeded the following cut-offs: fasting plasma glucose (FPG) ≥ 5.3mmol/l, 1-hour plasma glucose ≥ 10.0mmol/l, or 2-hour plasma glucose ≥ 8.6mmol/l [22]. A repeat OGTT at 24–28 weeks’ gestation (OGTT2) was prescribed to women with normal glucose values in OGTT1. 1105 women underwent OGTT2 at 22.2–34.0 weeks’ gestation, 81 women had missing results. The above-mentioned diagnostic thresholds were used to diagnose both early-onset GDM at OGTT1 and late-onset GDM at OGTT2 [22]. After receiving a GDM diagnosis at either time point, women were given diet and exercise guidance and advised to measure capillary glucose using regular finger-prick tests. If elevated glucose levels were recorded repeatedly (i.e., fasting glucose ≥ 5.5mmol/l or ≥ 7.8mmol/l 1 h after a meal), metformin, NPH insulin, or both treatments were prescribed.

Because evidence-based, internationally accepted OGTT criteria for the diagnosis of early-onset GDM are not available [23, 24], in the present study, we focused on women (*n* = 52) who required pharmacological GDM treatment at any point during pregnancy for persistent hyperglycemia in home-monitoring of blood glucose after an abnormal OGTT1 or OGTT2 result. Of these women with pharmacologically-treated GDM, 39 were diagnosed based on OGTT1 and 13 women based on OGTT2.

All laboratory analyses were centralized at the SKCH laboratory. The only exception was fasting serum insulin, which was analyzed at Vita Laboratoriot Oy, Helsinki, Finland. Plasma glucose during OGTTs was analyzed from 2mL fresh venous blood samples, drawn into citrate-fluoride blood collection tubes. Shortly after the blood draw, whole blood was separated into packed red cells, buffy coat, and plasma by centrifuging at 2540 RCF for 15 min at room temperature. Plasma glucose was determined with a photometric hexokinase method (Siemens Advia 1800 analyzer) within 5 h from the time of venipuncture. HbA_1c_ was analyzed from fresh venous blood samples, drawn into K2-EDTA blood collection tubes, using a quantitative latex agglutination inhibition method (Siemens Advia 1800 analyzer), in line with the recommendations of the International Federation of Clinical Chemistry. Fasting serum insulin was analyzed from venous blood samples drawn in conjunction with OGTT1. For these analyses, whole blood was centrifuged at 2540 RCF for 15 min at 4 °C and initially frozen at -80 C in aliquots of 1mL. The samples were thawed once for the analysis of insulin concentrations by the electrochemiluminescence immunoassay (ECLIA) method. Homeostatic Model Assessment for Insulin Resistance (HOMA-IR) was calculated using the following formula: fasting plasma glucose (mmol/l) times fasting serum insulin (mU/l) divided by 22.5.

Data are presented as means with standard deviation (SD) and as frequencies with percentages. Statistical comparisons between groups were done using the t-test or the chi-square test. The possible non-linear relationships between HbA_1c_ and plasma glucose values were modeled using restricted cubic splines regression models with 4 knots at the 5th, 35th, 65th, and 95th percentiles. Knot locations were based on Harrell’s [25] recommended percentiles. Adjustments were made for pre-gestational BMI and family history of type 2 diabetes. The accuracy of GDM risk factors and HbA_1c_ values in the prediction of plasma glucose concentrations > 90th percentile was evaluated with AUC (area under the curve), sensitivity, specificity, positive and negative predictive values, and likelihood ratio; 95% confidence intervals were obtained by bias-corrected bootstrapping (5000 replications). Differences between the AUCs were evaluated using an algorithm by DeLong. We defined the best cutoff value as the value with the highest accuracy that maximizes the Youden’s index. In the case of violation of the assumptions (e.g., non-normality) for continuous variables, a bootstrap-type method or Monte Carlo p-values (small number of observations) for categorical variables were used. Normal distributions were evaluated graphically and with the Shapiro–Wilk W-test. Stata 17.0 (StataCorp LP, College Station, TX, USA) was used for the analysis.

## Results

In our population-based cohort, the mean (SD) HbA_1c_ concentration (mmol/mol) at 8–14 weeks’ gestation was 33.6 (3.39). The highest HbA_1c_ value observed in our study population was 47 mmol/mol, i.e., we did not observe any women with an HbA_1c_ concentration ≥48 mmol/mol (≥6.5%) diagnostic of overt diabetes. The median time between early-pregnancy HbA_1c_ measurement and OGTT1 was 21 days (interquartile range 15 to 24). The mean (SD) HbA_1c_ (mmol/mol) at 8–14 weeks in women who fulfilled the FCCG criteria for early-onset GDM in OGTT1 (*n* = 208) was 35.3 (0.25), whereas, in women with a normal OGTT1 result according to the FCCG (*n* = 1186), the mean (SD) HbA_1c_ was 33.3 (0.09) (*p* < 0.001). For comparison, when applying the IADPSG OGTT criteria, the mean (SD) HbA_1c_ at 8–14 weeks was 34.8 (0.17) in those with an abnormal OGTT1 (*n* = 395), and 33.1 (0.10) in those with a normal OGTT1 (*n* = 999) (*p* < 0.001).

In OGTT1 at 12–16 weeks’ gestation, the mean (SD) plasma glucose concentrations (mmol/L) at 0 h, 1 h, and 2 h timepoints were 4.85 (0.33), 6.63 (1.73), and 5.60 (1.29), respectively. Plasma glucose concentrations ≥ 5.3, ≥ 8.9, and ≥ 7.3 mmol/L at OGTT1 corresponded to > 90th percentile at 0 h, 1 and 2 h timepoints, respectively. No cases of overt (type 1/type 2) diabetes were detected based on OGTT1 results.

Table 1 shows the basic maternal characteristics of women with at least one glucose concentration > 90th percentile (*n* = 308) and women with all glucose concentrations ≤ 90th percentile (*n* = 1086) in OGTT1 at 12–16 weeks’ gestation. Women with at least one glucose concentration > 90th percentile were older and more often parous and had more often a history of GDM in a previous pregnancy and/or a family history of type 2 diabetes compared to women with all plasma glucose concentrations ≤ 90th percentile in OGTT1. Women with plasma glucose concentrations > 90th percentile in OGTT1 were also characterized by higher mean pre-pregnancy weight and BMI, waist circumference, waist-to-height ratio (WHtR), fasting insulin concentration, HOMA-IR index, HbA_1c,_ and a higher rate of pharmacologically treated GDM.

**Table 1:** Maternal characteristics of 1394 southeastern Finnish women with singleton pregnancies divided into two groups based on 2 h 75 g oral glucose tolerance test results at 12 to 16 weeks’ gestation (OGTT1): women with one or more plasma glucose concentrations > 90th percentile and women with all plasma glucose concentrations (0 h, 1 h, and 2 h) ≤ 90th percentile

Maternal characteristic		Plasma glucose at OGTT1			p value
		≤ 90th percentile		> 90th percentile	
		*n* = 1086		*n* = 308	
Age (years), mean (SD)		29 (5)		31 (5)	< 0.001
Nulliparous, n (%)		580 (53)		127 (41)	< 0.001
Previous history of GDM, n (%)		44 (4)		74 (24)	< 0.001
Family history of type 2 diabetes, n (%)		450 (41)		149 (48)	0.030
Smoking during pregnancy, n (%)		112 (10)		31 (10)	0.87
Polycystic ovary syndrome, n (%)		58 (5)		22 (7)	0.23
Pre-gestational weight (kg), mean (SD)		68 (14)		78 (18)	< 0.001
Height (cm), mean (SD)		166 (6)		165 (6)	0.063
Pre-gestational BMI (kg/m2), mean (SD)		24.7 (4.6)		28.5 (6.5)	< 0.001
Waist circumference* (cm), mean (SD)		83 (10)		92 (12)	< 0.001
Waist-to-height ratio, mean (SD)		0.50 (0.06)		0.56 (0.08)	< 0.001
Early-pregnancy HbA_1c_* (mmol/mol), mean (SD)		33.1 (3.2)		35.1 (3.5)	< 0.001
Fasting insulin** (mU/L), mean (SD)		7.9 (6.8)		12.0 (12.0)	< 0.001
Insulin resistance index (HOMA-IR), mean (SD)		1.69 (1.45)		2.77 (2.79)	< 0.001
Pharmacologically-treated GDM, n (%)		9 (1)		43 (14)	< 0.001

*Measured at 8–14 weeks’ gestation

**Measured at 12–16 weeks’ gestation

**Fig. 1:**
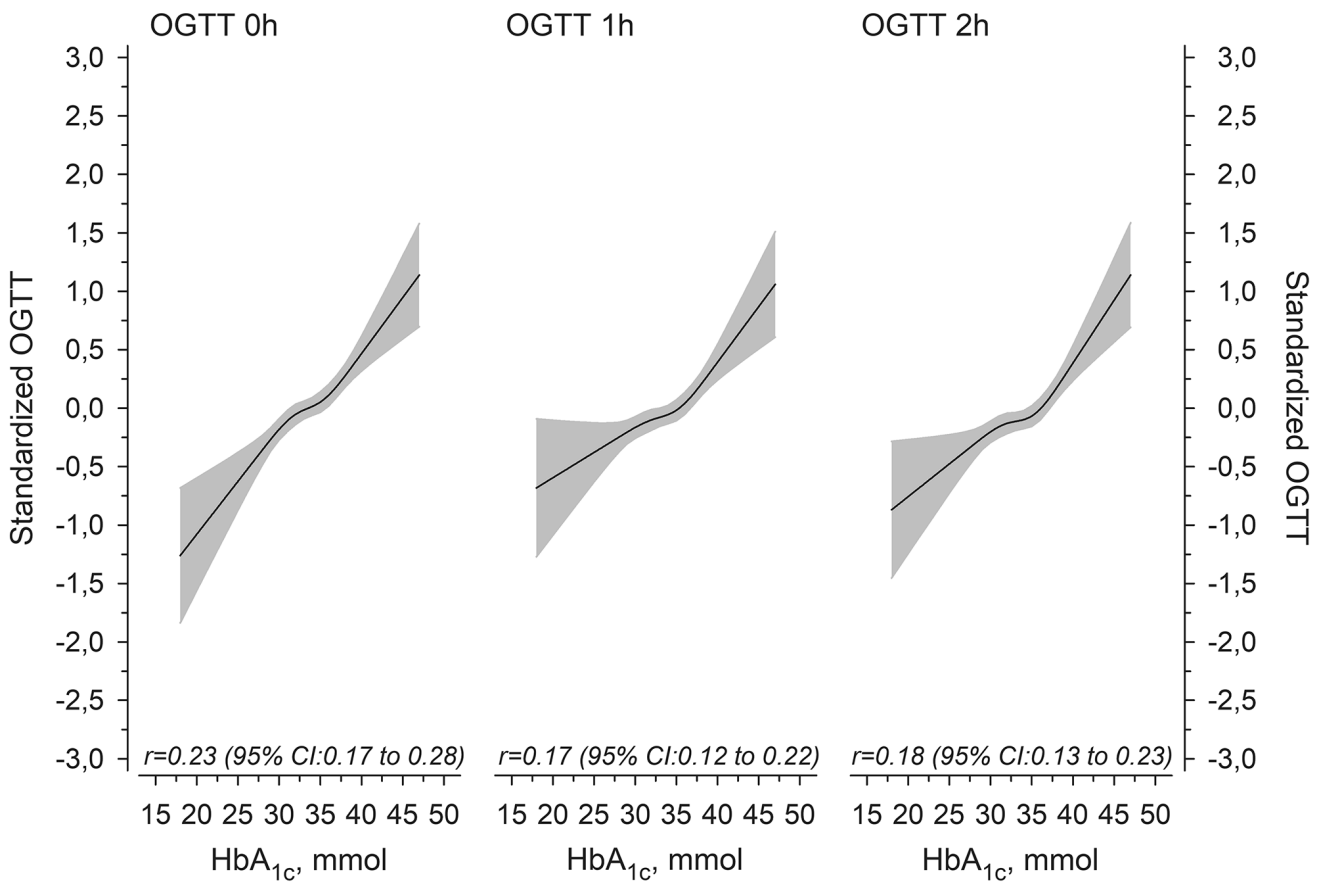
Early-pregnancy glycated hemoglobin (HbA_1c_) in relation to standardized values of plasma glucose concentrations at 0 h, 1 and 2 h timepoints of a 2-h 75 g oral glucose tolerance test at 12 to 16 weeks’ gestation in 1394 Finnish women. The results were adjusted for pre-gestational BMI and family history of type 2 diabetes

Figure 1 shows the relationships between HbA_1c_ measured at 8–14 weeks’ gestation and plasma glucose concentrations at 0 h, 1 h, and 2 h timepoints of an OGTT at 12 to 16 weeks’ gestation. An inflection point is observed around HbA_1c_ 35mmol/mol, after which the early-pregnancy HbA_1c_ concentration correlates positively with both fasting and post-load glucose concentrations during OGTT1. These analyses were adjusted for pre-pregnancy BMI and family history of type 2 diabetes.

**Fig. 2:**
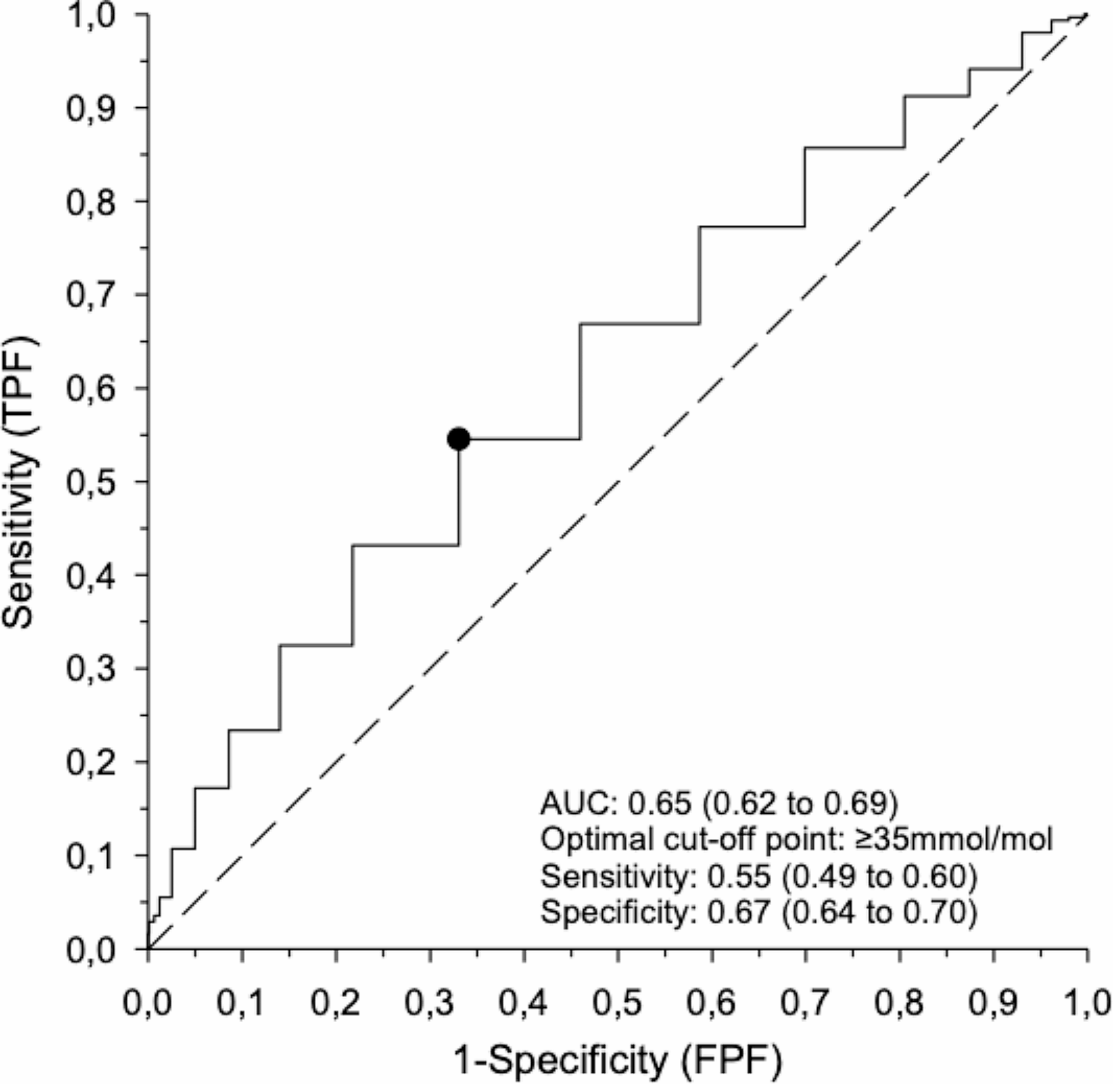
Receiver operating curve (ROC) analysis for diagnostic accuracy of glycated hemoglobin (HbA1c) at 8 to 14 weeks’ gestation to predict plasma glucose concentrations > 90 percentile at one or more timepoints (0 h, 1 h and/or 2 h) during an oral glucose tolerance test performed at 12 to 16 weeks’ gestation. The true positive fraction (TPF) is presented in the y-axis and the false negative fraction (FNF) in the x-axis. The black dot depicts the optimal cut-off point. Dotted line represents 45-degree reference line

Table 2 shows the best area-under-the-curve (AUC) cut-offs as well as sensitivity and specificity values for early-pregnancy HbA_1c_, maternal anamnestic risk factors (previous gestational diabetes, family history of type 2 diabetes, and history of polycystic ovary syndrome), maternal age, and maternal anthropometric parameters (pre-pregnancy BMI, WC, and WHtR) in the prediction of plasma glucose concentrations > 90th percentile at any timepoint of OGTT1 at 12 to 16 weeks’ gestation. Early-pregnancy HbA_1c_ ≥ 35 mmol/mol, i.e., the inflection point identified in Fig. 1 above, emerged as the best cut-off to predict high plasma glucose concentrations in OGTT1 (Fig. 2). Nevertheless, its AUC was 0.65, i.e., comparable to those of maternal anamnestic risk factors and maternal age > 30 years (Table 2). Maternal WC and WHtR (AUC 0.71 for both) performed better in the prediction of OGTT1 glucose concentrations > 90th percentile. Maternal WC, WHtR, BMI, and previous GDM were associated with the highest likelihood ratios and odds ratios.

**Table 2:** Prediction of plasma glucose concentrations > 90th percentile in a 2 h 75 g OGTT at 12 to 16 weeks’ gestation in 1394 Finnish women using risk factors (previous gestational diabetes, family history of type 2 diabetes, and history of polycystic ovary syndrome) and the best area under the curve (AUC) cut-offs for maternal age, body mass index (BMI), waist circumference, waist-to-height ratio, and glycated hemoglobin (HbA_1c_) measured at 8–14 weeks’ gestation

Risk factor	Best cut-off	AUC	Sensitivity	Specificity	Likelihood ratio	Odds ratio
Early-pregnancy HbA_1C_, mmol/mol	≥ 35	0.65 (0.62 to 0.69)	0.55 (0.49 to 0.60)	0.67 (0.64 to 0.70)	1.65 (1.45 to 1.88)	2.43 (1.88 to 3.14)
Previous gestational diabetes	Yes	0.60 (0.58 to 0.62)	0.24 (0.19 to 0.29)	0.96 (0.95 to 0.97)	5.93 (4.17 to 8.42)	7.49 (5.03 to 11.14)
Family history of type 2 diabetes	Yes	0.53 (0.50 to 0.57)	0.48 (0.43 to 0.54)	0.59 (0.56 to 0.62)	1.17 (1.02 to 1.34)	1.32 (1.03 to 1.71)
Polycystic ovary syndrome	Yes	0.51 (0.49 to 0.52)	0.07 (0.05 to 0.11)	0.95 (0.93 to 0.96)	1.34 (0.83 to 2.15)	1.36 (0.82 to 2.26)
Maternal age, years	≥ 30	0.62 (0.58 to 0.65)	0.66 (0.60 to 0.71)	0.52 (0.49 to 0.55)	1.37 (1.24 to 1.52)	2.09 (1.61 to 2.72)
Pre-gestational BMI, kg/m^2^	≥ 26.4	0.68 (0.65 to 0.72)	0.56 (0.51 to 0.62)	0.73 (0.70 to 0.75)	2.07 (1.81 to 2.38)	3.47 (2.67 to 4.50)
Waist circumference, cm	≥ 89	0.71 (0.67 to 0.74)	0.57 (0.51 to 0.62)	0.76 (0.73 to 0.79)	2.37 (2.05 to 2.74)	4.16 (3.18 to 5.44)
Waist-to-height ratio	≥ 0.54	0.71 (0.68 to 0.75)	0.56 (0.50 to 0.61)	0.78 (0.75 to 0.80)	2.51 (2.16 to 2.93)	4.42 (3.37 to 5.79)

To investigate whether the ability of HbA_1c_ to predict high OGTT1 glucose concentrations improves in the presence of other maternal risk characteristics, we repeated our analyses combining HbA_1c_ ≥ 35 mmol/mol with each of the maternal anthropometric or anamnestic risk factors shown in Table 3. However, no improvement in the predictive accuracy of HbA_1c_ ≥ 35 mmol/mol was detected. Maternal anthropometric variables (BMI, WC, WHtR), in particular, performed better alone (Table 2) than in combination with HbA_1c_ ≥ 35 mmol/mol (Table 3) in the prediction of OGTT1 glucose concentrations > 90th percentile.

**Table 3:** The accuracy of early-pregnancy glycated hemoglobin (HbA_1c_) ≥ 35mmol/mol in combination with other maternal GDM risk factors in the prediction plasma glucose concentrations > 90th percentile at one or more time points (0 h, 1 h, and/or 2 h) of a 2 h 75 g OGTT at 12 to 16 weeks gestation in 1394 Finnish women. The best area under the curve (AUC) cut-offs for pre-pregnancy body mass index (BMI), waist circumference at 8–14 weeks’ gestation, and waist-to-height ratio at 8–14 weeks’ gestation to predict plasma glucose concentrations > 90th percentile in an OGTT at 12 to 16 weeks’ gestation alone without HbA_1c_ (see Table 2) are indicated with an asterisk (*)

Maternal risk factor in addition to HbA_1c_ ≥ 35 mmol/mol	AUC	Sensitivity	Specificity	Likelihood ratio	Odds ratio
Previous gestational diabetes	0.57 (0.55 to 0.59)	0.15 (0.11 to 0.20)	0.98 (0.97 to 0.99)	9.21 (5.43 to 15.62)	10.68 (6.14 to 18.59)
Family history of type 2 diabetes	0.56 (0.54 to 0.59)	0.26 (0.21 to 0.32)	0.86 (0.84 to 0.88)	1.90 (1.50 to 2.42)	2.23 (1.64 to 3.02)
Polycystic ovary syndrome	0.51 (0.50 to 0.52)	0.04 (0.02 to 0.07)	0.98 (0.97 to 0.99)	2.29 (1.15 to 4.55)	2.35 (1.17 to 4.72)
Maternal age ≥ 30 years	0.61 (0.58 to 0.64)	0.40 (0.34 to 0.45)	0.83 (0.80 to 0.85)	2.29 (1.89 to 2.77)	3.13 (2.38 to 4.13)
Pre-gestational BMI, kg/m^2^					
≥25	0.61 (0.58 to 0.63)	0.35 (0.29 to 0.40)	0.87 (0.84 to 0.89)	2.58 (2.08 to 3.20)	3.43 (2.56 to 4.59)
≥26.4*	0.61 (0.58 to 0.63)	0.30 (0.25 to 0.36)	0.91 (0.89 to 0.93)	3.35 (2.60 to 4.31)	4.36 (3.17 to 6.00)
≥30	0.59 (0.56 to 0.61)	0.22 (0.18 to 0.27)	0.96 (0.94 to 0.97)	5.10 (3.60 to 7.24)	6.26 (4.22 to 9.30)
≥35	0.55 (0.53 to 0.56)	0.11 (0.07 to 0.15)	0.98 (0.97 to 0.99)	6.46 (3.69 to 11.32)	7.12 (3.98 to 12.74)
Waist circumference, cm					
≥80	0.63 (0.60 to 0.66)	0.48 (0.42 to 0.53)	0.79 (0.76 to 0.81)	2.25 (1.90 to 2.65)	3.38 (2.58 to 4.43)
≥88	0.62 (0.59 to 0.65)	0.34 (0.29 to 0.40)	0.90 (0.88 to 0.92)	3.41 (2.68 to 4.34)	4.64 (3.40 to 6.35)
≥89*	0.62 (0.59 to 0.65)	0.32 (0.27 to 0.38)	0.92 (0.90 to 0.93)	3.79 (2.94 to 4.89)	5.12 (3.72 to 7.05)
≥90	0.62 (0.59 to 0.65)	0.32 (0.27 to 0.37)	0.92 (0.90 to 0.94)	4.11 (3.15 to 5.36)	5.56 (3.99 to 7.74)
Waist-to-height ratio					
≥0.50	0.63 (0.60 to 0.66)	0.42 (0.36 to 0.48)	0.84 (0.82 to 0.86)	2.64 (2.17 to 3.20)	3.82 (2.88 to 5.07)
≥0.54*	0.62 (0.59 to 0.65)	0.31 (0.26 to 0.37)	0.92 (0.91 to 0.94)	4.07 (3.13 to 5.30)	5.48 (3.95 to 7.60)

Although HbA_1c_ ≥ 35mmol/mol alone or in combination with other maternal risk factors did not effectively predict plasma glucose values > 90th percentile in OGTT1, its diagnostic accuracy was better with respect to prediction of pharmacologically treated GDM (diagnosed at OGTT1 or OGTT2): AUC (95% CI) 0.75 (0.68 to 0.81), sensitivity 0.75 (0.61 to 0.86), specificity 0.64 (0.61 to 0.66). HbA_1c_ threshold ≥ 35mmol/mol detected 39/52 (75%), whereas a HbA_1c_ threshold ≥ 39mmol/mol detected 14/52 (27%) women with pharmacologically treated GDM. The lowest HbA_1c_ concentrations in early pregnancy preceding OGTT1 plasma glucose concentrations > 90th percentile or pharmacologically treated GDM were 24 and 29 mmol/mol, respectively.

## Discussion

In the population-based EDDIE cohort, maternal HbA_1c_ recorded at 8–14 weeks’ gestation was associated with maternal plasma glucose concentrations in a 2 h 75 g OGTT at 12–16 weeks’ gestation. In ROC curve analysis, early-pregnancy HbA_1c_ ≥ 35mmol/mol was the best cut-off for the prediction of pharmacologically-treated GDM diagnosed at any point during pregnancy or plasma glucose concentrations > 90th percentile in the OGTT at 12–16 weeks. However, the diagnostic accuracy of HbA_1c_ ≥ 35mmol/mol in the prediction of plasma glucose concentrations over > 90th percentile in an early-pregnancy OGTT was limited both alone and in combination with established maternal risk factors (previous GDM, family history of type 2 diabetes, or increased pre-pregnancy BMI, maternal age, waist circumference, or waist-to-height ratio).

To the authors’ knowledge, our study is the largest study to date to examine early-pregnancy HbA_1c_ concentrations and the associations and interactions between maternal HbA_1c_ and plasma glucose concentrations in an OGTT at 12–16 weeks’ gestation, and among the only studies utilizing a population-based cohort. While some previous studies have observed altered glucose dynamics [18] and increased frequencies of early-onset GDM [19] in women with elevated HbA_1c_ levels in early pregnancy, others have reported poor diagnostic accuracy for early HbA_1c_ testing in the prediction of early-onset GDM [16, 17, 20]. These discrepancies could be, for example, due to small sample sizes [18], low participation in early-pregnancy OGTTs [16], and HbA_1c_ measurements near mid-pregnancy [17, 20]. Furthermore, the interpretation of study results has been complicated due to the current lack of consensus on the best diagnostic thresholds for “early-onset” GDM [23, 24]. Considering that no evidence-based OGTT cut-offs for early GDM are available, the fact that we were able to analyze plasma glucose concentrations as continuous variables in all women is an advantage. The EDDIE sample size is robust and pre-existing diabetes was reliably excluded with HbA_1c_ testing and OGTTs that were completed by all participants. All OGTTs were performed in the same laboratory, utilizing standard subject preparations and test protocol. Among the limitations of the study is the ethnically homogenous Caucasian study population, which may affect the generalizability of our results to other ethnic groups. Furthermore, we used self-reported data on family history of type 2 diabetes and polycystic ovary syndrome, which may result in some recall bias. Of note, the iron status of the women in our cohort was not determined, which could potentially influence HbA_1c_ results [26].

Interestingly, none of the women in our Finnish obstetric study population had early-pregnancy HbA_1c_ concentrations fulfilling the criteria for overt diabetes, i.e. ≥48 mmol/mol (≥6.5%) and no new type 1 or type 2 diabetes diagnoses were made based on the HbA_1c_ screening. In comparison, in their large study in New Zealand, Hughes et al. observed early-pregnancy HbA_1c_ ≥48 mmol/mol in 0.2% of women [16]. Ethnicity-related metabolic differences or differences related to the effectiveness of health care systems to identify overt diabetes cases before pregnancy might be behind these discrepancies in our findings.

Due to physiological changes that occur in maternal glucose metabolism across gestation, it is not expectable that the same OGTT diagnostic criteria are ideal for the diagnosis of GDM in early- and late pregnancy [21]. Considering that neither the FCCG nor the IADPSG criteria have been validated for the diagnosis of early-onset GDM, we exploited our population-based setting and defined plasma glucose concentrations > 90th percentile as “early pregnancy hyperglycemia”. The maternal characteristics shown in Table 1 support an adverse metabolic profile in women with OGTT glucose concentrations > 90th percentile. The mean HbA_1c_ levels in women with OGTT1 glucose concentrations ≤ 90th percentile was comparable to that observed in women who were normoglycemic as assessed by the FCCG or the IADPSG criteria (∼33 mmol/mol in all groups). On the other hand, in women with OGTT1 glucose concentrations > 90th percentile, the mean HbA_1c_ was comparable to the mean HbA_1c_ concentrations recorded in women with early-onset GDM diagnosed with the FCCG or IADPSG criteria (∼35 mmol//mol in all groups). Moreover, women with OGTT1 glucose concentrations > 90th percentile were characterized by adiposity by every assessed parameter (BMI, WC, WHtR, weight) and had higher fasting insulin levels and HOMA-IR indices than women with OGTT1 glucose concentrations ≤ 90th percentile. They were also older, in accordance with the fact that weight gain, development of central obesity [27], and increase in plasma glucose concentrations [28] typically occur with aging.

The relationship between maternal HbA_1c_ and late-onset GDM has been studied extensively. In general, the higher the maternal HbA_1c_ concentration in early or late pregnancy, the higher the prevalence of GDM in mid- or late pregnancy [26]. Previously, different HbA_1c_ thresholds from 36 to 42 mmol/mol have been suggested to best predict late-onset GDM (reviewed by Renz [14] and Kattini [15] et al.), but the sensitivity of all of these previously suggested thresholds has been found to be relatively low and a considerable number of women with late-onset GDM have HbA_1c_ concentrations within the normal range [26]. Less is known about the concordance of HbA_1c_ and OGTT results in early pregnancy. A Norwegian study on 677 low-risk women suggested that mid-pregnancy HbA_1c,_ measured at 18 to 22 weeks’ gestation does not predict GDM diagnosed with a 75 g 2 h OGTT at the same time point in pregnancy, using the WHO 1999 or modified IADPSG criteria [17]. Hughes et al., on the other hand, found HbA_1c_ ≥ 41mmol/mol at a median 6.7 weeks’ gestation to have a high specificity (98.4%) but poor sensitivity (18.8%) in the prediction of GDM < 20 weeks’ gestation in an unselected population (*n* = 974) utilizing the IADPSG criteria [16]. These previous reports in low-risk/unselected populations are in line with the present findings in our larger population-based cohort suggesting that HbA_1c_ is positively associated with early-pregnancy fasting and post-load glucose concentrations but shows limited utility in the prediction of early-pregnancy hyperglycemia.

Inconsistent results have also been reported by studies performed in higher-risk obstetric populations. Bozkurt et al. concluded that women who had been referred to a tertiary center with an HbA_1c_ ≥ 39mmol/mol (*n* = 23) had more early-onset GDM utilizing the IADPSG criteria, higher plasma glucose concentrations in an 2-h 75 g OGTT, and characteristics suggesting beta-cell dysfunction at median 16 weeks’ gestation, compared to women with HbA_1c_ < 39mmol/mol (*n* = 197) [18]. Another small study (*n* = 243), by Battarbee et al., involving women with prior GDM or obesity, found an association between elevated HbA_1c_ and GDM < 21 weeks’ gestation: Women with early GDM (*n* = 14) had higher HbA_1c_ levels compared to women without GDM (*n* = 229) [19]. In this study, the AUC for HbA_1c_ in the prediction of early-onset GDM (diagnosed with 2-step testing) was 0.80, with 64% sensitivity, and 84% specificity for an HbA_1c_ threshold of 5.6% (38 mmol/mol). In contrast, in a larger study (*n* = 869) by Immanuel et al. reported that HbA_1c_ was a poor predictor of GDM (at any timepoint of pregnancy, also < 20 weeks’ gestation, in women of European (mostly Caucasian) origin with BMI ≥ 29 kg/m^2^ [20]. The observations of Immanuel et al. are in agreement with our analyses combining early-pregnancy HbA_1c_ and various maternal risk factors, including obesity, which suggested that even in selected high-risk groups the diagnostic accuracy of early-pregnancy HbA_1c_ in the prediction of high OGTT glucose concentrations is limited.

Previous studies have identified early-pregnancy elevation of HbA_1c_ to be a risk factor for pharmacological treatment of high glucose levels during pregnancy [29, 30] and adverse maternal and neonatal outcomes even without increased fasting or post-load glucose concentrations diagnostic for GDM [29, 31]. This is consistent with our results showing that HbA_1c_ ≥ 35mmol/mol at 8–14 weeks’ gestation predicts pharmacologically treated GDM diagnosed at any point during pregnancy. Women who require pharmacological treatment for GDM have had persistent abnormal capillary glucose levels in the home monitoring of blood glucose, despite diet treatment, and hence these women are likely to be characterized by more severe defects in glucose metabolism. It is possible that early-pregnancy HbA_1c_ ≥ 35mmol/mol could be used in the early identification of women with an increased risk of more severe GDM subtypes, but this should be further investigated in larger and more diverse study populations.

## Conclusions

To summarize, the results of our population-based study suggest that maternal HbA_1c_ ≥ 35mmol/mol recorded at 8–14 weeks’ gestation is positively associated with plasma glucose concentrations in a 2 h 75 g OGTT at 12–16 weeks’ gestation, although its diagnostic accuracy in the prediction of high plasma glucose concentrations over > 90th percentile in an early-pregnancy OGTT was poor both alone and in combination with maternal anamnestic and anthropometric risk factors. However, early-pregnancy HbA_1c_ ≥ 35mmol/mol predicted pharmacologically treated GDM diagnosed at any point during pregnancy, suggesting potential utility in first-trimester identification of parturients at risk of developing severe GDM subtypes.

### Electronic supplementary material

Below is the link to the electronic supplementary material.

Supplementary Figure 1: A flow chart depicting the formation of the Early Diagnosis of Diabetes in Pregnancy (EDDIE) study population for the present study.

## Data Availability

The datasets generated and analyzed during the current study are not publicly available due to reasons of sensitivity but may be available from the corresponding author on reasonable request.

## Abbreviations

GDM: Gestational diabetes
HbA_1c_: Glycated hemoglobin A_1c_
IADPSG: International Association of Diabetic Pregnancy Study Groups
OGTT: Oral glucose tolerance test
WC: Waist circumference
WHtR: Waist-to-Height-Ratio
