# Supplementary figures and images for: First-trimester HbA1c in relation to plasma glucose concentrations in an oral glucose tolerance test at 12 to 16 weeks’ gestation—a population-based study
Source: Diabetol Metab Syndr. 2024 Feb 27;16:53. doi: 10.1186/s13098-024-01290-3 (PMC10898079; doi:10.1186/s13098-024-01290-3)

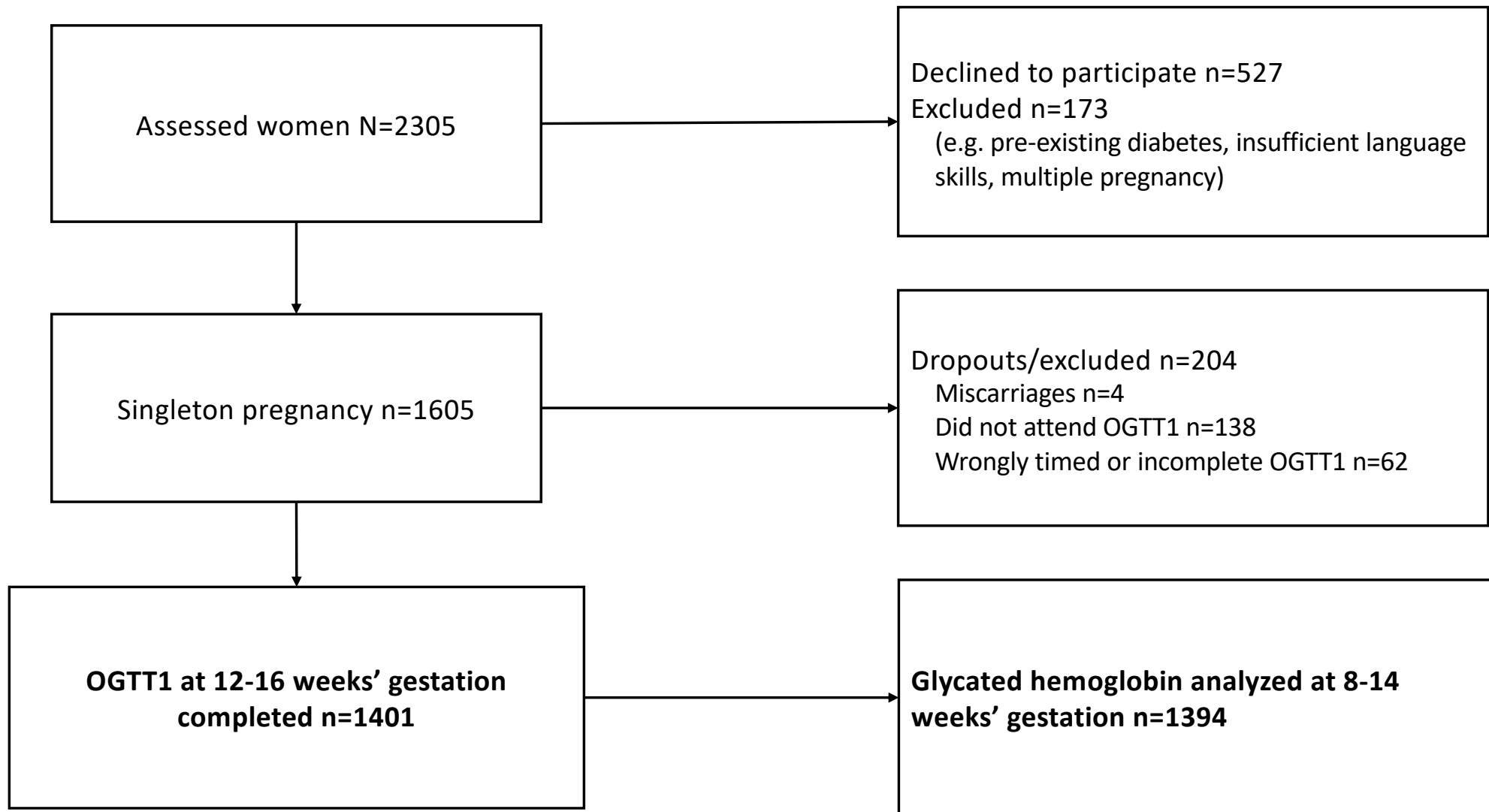

Supplement: Supplementary file 1 — Supplementary Figure 1: A flow chart depicting the formation of the Early Diagnosis of Diabetes in Pregnancy (EDDIE) study population for the present study. [file 13098_2024_1290_MOESM1_ESM.pdf]
